# Molecular characterization and zoonotic potential of *Giardia* and *Cryptosporidium* infections in dogs and cats in Central Spain

**DOI:** 10.1016/j.fawpar.2026.e00351

**Published:** 2026-06-17

**Authors:** Juan P. Barrera, Ana Montoya, Aida de Lucio, Pamela C. Köster, Begoña Bailo, David Carmena, Guadalupe Miró

**Affiliations:** a Department of Animal Health, Faculty of Veterinary, Complutense University of Madrid, Madrid, Spain; b Faculty of Health Sciences, Alfonso X El Sabio University (UAX), Villanueva de la Cañada, Spain; c Parasitology Reference and Research Laboratory, Spanish National Centre for Microbiology, Health Institute Carlos III, Majadahonda, Spain; d Center for Biomedical Research Network in Infectious Diseases (CIBERINFEC), Health Institute Carlos III, Madrid, Spain

**Keywords:** Molecular epidemiology, Zoonosis, ssu rRNA, Gdh, Bg, gp60

## Abstract

*Giardia duodenalis* and *Cryptosporidium* spp. are environmentally resistant protozoan parasites transmitted via the faecal-oral route, often through contaminated water or food. Although companion animals may contribute to environmental dissemination of infective stages, their role in zoonotic transmission remains debated. In this study, 75 *Giardia*-positive (65 dogs, 10 cats) and 20 *Cryptosporidium*-positive (11 dogs, 9 cats) faecal samples from central Spain were molecularly characterized at the *gdh* and *bg* loci for *G. duodenalis* and at the *ssu* rRNA and *gp60* loci for *Cryptosporidium* spp. Host-adapted *Giardia* assemblages predominated, with assemblages C (33.9%) and D (47.7%) in dogs and assemblage F (50.0%) in cats, while zoonotic assemblages A and B occurred at moderate frequencies (10.8%–40.0%). *Cryptosporidium canis* and *Cryptosporidium felis* were the predominant species in dogs and cats, respectively, whereas *C. parvum* and *C. hominis* were sporadically detected. These findings expand molecular data on enteric protozoa in companion animals in Spain and highlight their potential relevance within a One Health context.

## Highlights

• Host-adapted *Giardia* and *Cryptosporidium* lineages predominated in dogs and cats.
• Zoonotic *Giardia* assemblages A and B were detected in both host species.
• Anthroponotic *C. hominis* and zoonotic *C. parvum* were sporadically detected.
• *Cryptosporidium canis* subtype XXe2 was identified in a dog.

## Graphical abstract

**Figure:**
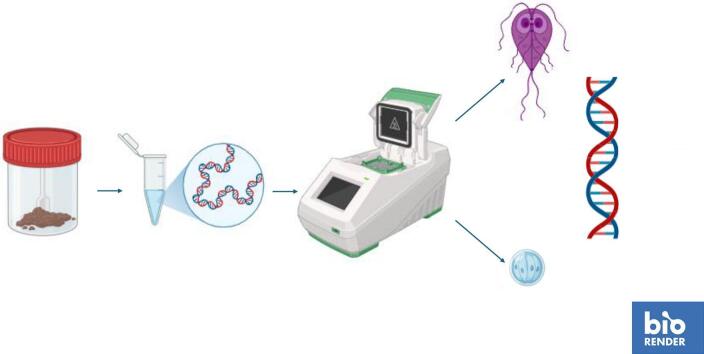

## 1 Introduction

Enteric protozoan parasites of the genera *Giardia* and *Cryptosporidium* are common causes of gastrointestinal disease in humans and a wide range of mammals, including dogs and cats. In companion animals, particularly puppies, kittens, geriatric and immunocompromised individuals are considered especially susceptible to these infections (Scorza and Tangtrongsup, 2010; Tangtrongsup and Scorza, 2010; Tysnes et al., 2014). Transmission occurs mainly through the faecal-oral route, either indirectly via ingestion of infective stages (cysts and oocysts) present in contaminated water, food, or fomites, or directly through contact with faecally contaminated environments. Owing to their environmental resistance, both parasites can persist in water and moist conditions, facilitating their transmission and their involvement in water and foodborne outbreaks worldwide, representing a significant public health concern.

*Giardia duodenalis* is the species complex responsible for giardiosis in human and a broad range of mammals, including dogs and cats (Adam, 2021). It comprises eight genetically distinct assemblages (A-H) that differ in host range and zoonotic potential (Ryan et al., 2021). Assemblages A and B exhibit a wide host specificity and are considered zoonotic (Cai et al., 2021), whereas assemblages C and D are mainly adapted to canids, assemblage E to wild and domestic ungulates, assemblage F to felids, assemblage G to rodents and assemblage H to marine pinnipeds (Ryan et al., 2021). Based on their strong host adaption, assemblages C and D have been proposed to correspond to *Giardia canis*, whereas assemblage F has been proposed as *Giardia cati* (Wielinga et al., 2023).

At least 44 species and more than 120 genotypes of *Cryptosporidium* have been described. Among them, *Cryptosporidium canis* and *Cryptosporidium felis* are primarily adapted to infect canids and felids, respectively, although both species have occasionally been reported causing human infections (Ryan et al., 2021).

PCR-based methods coupled with Sanger sequencing remain essential tools for investigating the genetic diversity of *G. duodenalis* and *Cryptosporidium* spp. in epidemiological studies. The small subunit ribosomal RNA (*ssu* rRNA) gene is widely used for sensitive detection due to its multicopy nature (Jiang et al., 2005; Nantavisai et al., 2007). However, further discrimination within species requires more polymorphic markers, such as the glutamate dehydrogenase (*gdh*) and beta-giardin (*bg*) genes for *G. duodenalis* (Cacciò et al., 2002; Monis et al., 1996) and the 60-kDa glycoprotein (*gp60*) gene for *Cryptosporidium* spp. (Strong et al., 2000).

In Spain, molecular data on the genetic diversity of these parasites in canine and feline populations remain scarce. To date, only a limited number of geographically restricted studies have characterized *G. duodenalis* assemblages and *Cryptosporidium* infections in companion animals (Table S1 and Table S2). Although host-adapted variants appear to predominate, zoonotic species and genotypes have occasionally been detected, suggesting that dogs and cats could potentially cause water- or foodborne human infections through environmental contamination with infective (oo)cysts.

This study aimed to molecularly characterise *G. duodenalis* and *Cryptosporidium* spp. isolates from dogs and cats obtained in a previous epidemiological survey conducted in central Spain (Barrera et al., 2025), contributing new molecular data relevant to the understanding of potential transmission pathways at the animal-environment-human interface.

## 2 Materials and methods

### 2.1 Ethical statement

This study was carried out in accordance with Spanish legislation guidelines (RD 8/2003) and with the International Guiding Principles for Biomedical Research Involving Animals issued by the Council for International Organization of Medical Sciences and the International Council for Laboratory Animal Science (RD 53/2013).

### 2.2 Study design

This study was based on the molecular characterization of *G. duodenalis* and *Cryptosporidium* spp. isolates obtained from a previously described retrospective cohort comprising 15,899 faecal samples (10,813 from dogs and 5086 from cats) collected between 2013 and 2023 (Barrera et al., 2025). All samples were submitted to a reference veterinary diagnostic laboratory in Madrid (Spain), and therefore the study population reflects a geographically restricted dataset corresponding to the laboratory's service area. However, although samples originated from Madrid-based clinical submissions, individual animals may have travelled outside the region and in some cases may have originated from other Spanish autonomous communities, particularly in the case of shelter animals. This population mobility could potentially contribute to the genetic diversity observed in the *G. duodenalis* and *Cryptosporidium* sequences identified in this study.

The number of PCR analyses performed for each parasite depended on the diagnostic requests made by the attending veterinary clinicians. For *G. duodenalis* detection, PCR amplification was performed on a total of 634 cases, comprising 451 canine and 183 felines samples. For *Cryptosporidium* spp., a total of 407 PCR assays were carried out, distributed across 250 dogs and 157 cats. To ensure maximum diagnostic rigor and strictly avoid the reporting of false positives, all *Cryptosporidium* PCR-positive samples were systematically subjected to sequencing for species confirmation. Consequently, a sample was only definitively classified and recorded as positive for *Cryptosporidium* if its species identity was successfully verified through sequence analysis.

### 2.3 DNA extraction and purification

Canine and feline faecal concentrates with a positive result to *G. duodenalis* and *Cryptosporidium* spp. in any of the diagnostic methods used in Barrera et al. (2025) were included in this survey and shipped to the Parasitology Reference and Research Laboratory, National Centre for Microbiology, Majadahonda (Madrid) for downstream molecular testing. Genomic DNA was isolated from about 200 mg of each concentrated faecal sample using the QIAamp Fast DNA Stool Mini Kit (Qiagen, Hilden, Germany) according to the manufacturer's instructions and eluted in 200 μl of PCR-grade water.

### 2.4 Molecular confirmation and characterization of *Giardia duodenalis*

Confirmation of *G. duodenalis* infections was achieved using a real-time PCR (qPCR) method targeting the gene codifying the small subunit ribosomal RNA (*ssu* rRNA) of the parasite (Verweij et al., 2003).

For assessing the molecular diversity of the parasite at the assemblage and sub-assemblage levels, we used a semi-nested PCR to amplify a fragment of the *gdh* gene (Read et al., 2004) and nested PCR to amplify a fragment of the *bg* gene (Cacciò et al., 2002; Lalle et al., 2005).

### 2.5 Molecular confirmation and characterization of *Cryptosporidium* spp.

The presence of *Cryptosporidium* spp. was assessed using a nested-PCR protocol to amplify a fragment of the *ssu* rRNA gene of the parasite (Tiangtip and Jongwutiwes, 2002). Subtyping tools based on the amplification of partial sequences of the 60-kDa glycoprotein (*gp60*) gene were used to ascertain intra-species genetic diversity in samples that tested positive for *C. canis* (Jiang et al., 2021) and *C. hominis* or *C. parvum* (Feltus et al., 2006).

### 2.6 General PCR and electrophoretic procedures

Detailed information on the PCR cycling conditions and oligonucleotide sequences used for the molecular identification and/or characterization of the protozoan parasites investigated in the present study is presented in Table S3 and Table S4, respectively.

### 2.7 Sanger sequencing analyses

Positive-PCR products of the expected size were directly sequenced in both directions using appropriate internal primer sets (Table S4). DNA sequencing was conducted by capillary electrophoresis using the BigDye® Terminator chemistry on an on ABI PRISM 3130 automated DNA sequencer (Applied Biosystems). Generated DNA consensus sequences were aligned to appropriate reference sequences obtained in GenBank using the Basic Local Alignment Search Tool (BLAST) and the MEGA X software (Kumar et al., 2018) for species confirmation and genotype identification. Mixed *G. duodenalis* infections were inferred when sequence analyses at the two genotyping markers (*gdh* and *bg*) yielded discordant assemblage assignments. The sequences obtained in this study have been deposited in GenBank under the following accession numbers: PV135062– PV135105 and PV135041– PV135060 (*G. duodenalis*) and PV123153–PV123160 and PV135061 (*Cryptosporidium* spp.).

### 2.8 Phylogenetic analysis

To analyse the phylogenetic relationship among various subtype families of *C. canis*, a maximum-likelihood tree was constructed using the MEGA X software (Kumar et al., 2018), based on substitution rates calculated with the general time reversible model and gamma distribution with invariant sites (G + I). Bootstrapping with 1000 replicates was used to determine support for the clades (Jiang et al., 2021).

## 3 Results

Regarding *G. duodenalis* screening, out of 451 PCR assays performed on canine samples, 281 were positive, of which 65 were successfully genotyped, representing a sequencing success rate of 23.1% (65/281). In the feline population, 37 out of 183 PCR assays were positive for *G. duodenalis*, with 10 isolates successfully genotyped, yielding a success rate of 27.0% (10/37). For *Cryptosporidium* spp., 11 out of 250 canine PCR assays were positive and all isolates were successfully sequenced. Similarly, 9 out of 157 feline PCR assays were positive and successfully sequenced.

Overall, genotyping data were obtained from 75 *G. duodenalis* isolates (canine, *n* = 65; feline, *n* = 10) and 20 *Cryptosporidium* spp. isolates (canine, *n* = 11; feline, *n* = 9) from animals of different origins and ages.

### 3.1 Molecular characterization of *Giardia duodenalis* isolates

All *G. duodenalis* positive samples (*n* = 75) were confirmed by qPCR yielding cycle threshold (C_T_) values <32. Amplification success rates at the *gdh* and *bg* loci and assemblage distributions are summarized in Table 1.

**Table 1:** Sequence typing results of the *Giardia duodenalis*-positive samples of canine (*n* = 65) and feline (*n* = 10) origin successfully genotyped at any of the two loci.

Host species	*Assemblage*	*gdh* only	*bg* only	Both	Total
Dog	A	2	0	0	2
	B	3	0	0	3
	C	14	3	5	22
	D	16	3	12	31
	F	1	0	0	1
	B + C	0	0	1	1
	B + D	0	0	1	1
	C + D	2	0	1	3
	D+X^a^	1	0	0	1
**Sub-total**		**39**	**6**	**20**	**65**
Cat	A	2	0	0	2
	B	2	0	0	2
	D	1	0	0	1
	F	3	1	1	5
**Sub-total**		**8**	**1**	**1**	**10**

a Unknown assemblage due to low quality sequence data.

Fifty-five *G. duodenalis* isolates were successfully genotyped in dogs, revealing the presence of canine-adapted assemblages C (*n* = 20) and D (*n* = 28) and zoonotic assemblages A (*n* = 2) and B (*n* = 3). Mixed infections (*n* = 4) involving combinations of assemblages B, C and D were also inferred based on discordant assemblage assignments at different genotyping markers.

In cats, assemblages A (*n* = 2), B (*n* = 1), D (*n* = 1) and F were identified, with assemblage F (*n* = 4) being the most common. No mixed infections were observed among feline isolates.

Sub-assemblage diversity and nucleotide variation detected at the *gdh* locus and *bg* locus are summarized in Table 2 and Table 3, respectively.

**Table 2:** Frequency and molecular diversity of *Giardia duodenalis* identified at the *gdh* locus in the canine and feline populations (GenBank accession numbers are provided).

Host	Assemblage	Sub-assemblage	No. isolates	Reference sequence	Stretch	Single nucleotide polymorphisms	GenBank ID
Dog	A	AI	1	L40509	77–482	C142T, C447T	PV135062
		AI	1	L40509	123–444	C428T	PV135063
	B	BIII	1	AF069059	42–411	C87T, T147C, T186C, T219C, C309T, C336T, G402A	PV135064
		BIV	1	L40508	125–487	C179T, T183C, T387C, C396T, C423T, T429C	PV135065
		BIV	1	L40508	76–496	T183C, T387C, C396T, C423T	PV135066
	C	–	15	U60984	76–491	None	PV135067
		–	1	U60984	115–491	G193T	PV135068
		–	1	U60984	125–482	C207Y, T266W, C465Y	PV135069
		–	1	U60984	123–491	A224T	PV135070
		–	1	U60984	131–485	G276R	PV135071
		–	1	U60984	113–486	C423M	PV135072
	D	–	1	U60986	130–491	G153A, C314M	PV135073
		–	1	U60986	76–491	G153A, T429C, G441A, C461A	PV135074
		–	1	U60986	124–491	T159W, G201A, T210C, T429C, G441A	PV135075
		–	1	U60986	76–491	224DelA, T240C, T395C, T429C, G441A, T459A	PV135076
		–	1	U60986	67–491	T240C	PV135077
		–	1	U60986	124–486	T240C, C314M	PV135078
		–	1	U60986	76–491	T240C, C370Y	PV135079
		–	2	U60986	117–491	T240C, T429C, G441A, T459A	PV135080
		–	1	U60986	115–491	T240Y, G274S	PV135081
		–	1	U60986	111–491	T240Y, C314M, C375Y, T429Y, G441R	PV135082
		–	1	U60986	130–471	T240Y, C375Y	PV135083
		–	2	U60986	108–485	T240Y, T429Y, G441R	PV135084
		–	1	U60986	123–449	T312C	PV135085
		–	1	U60986	165–486	A322R, T324K, C375Y, T429Y, G441R	PV135086
		–	1	U60986	170–482	G369R, T429C, G441A	PV135087
		–	1	U60986	147–491	G369R, G441A	PV135088
		–	1	U60986	78–482	C375T	PV135089
		–	1	U60986	111–468	C375Y, T429Y, G441R	PV135090
		–	1	U60986	76–491	T429Y	PV135091
		–	2	U60986	78–480	T429C, G441A	PV135092
		–	1	U60986	76–496	T429C, G441A, C492T	PV135093
		–	1	U60986	75–491	C441T, T429C, G441A	PV135094
		–	3	U60986	–	Unknown	–
	F	–	1	AF069057	145–486	None	PV135095
	B + D	BIV	1	U60986	123–491	T126Y, T144Y, T159Y, A162R, T168Y, T171Y, C174Y, C198Y, T204Y, T210Y, C219Y, C222Y, T234K, T240Y, T246Y, C255Y, A261R, C273Y, T279Y, C294S, T303Y, C314M, T318Y, C321Y, A333M, C335S, C336Y, C345Y, C351Y, C360M, C372Y, C375Y, A390R, A393M, C401S, A426R, G441R, G450R, C453S, T459W, C462Y, T474K	PV135096
	C + D	–	1	U60986	115–482	G129R, T135Y, T150Y, T159Y, A162W, T168Y, T171Y, T210Y, T234Y, T240Y, T246Y, A261R, T267Y, T279Y	PV135097
		–	1	U60986	131–381	T135Y, T150Y, T159Y, A162W, T168Y, T171Y, T210Y, T234Y, T240C, T246Y, A261R, T267Y, T279Y, T303Y, T318Y, C327Y, A333M, C336Y, T348Y, C360S, A393R, T396Y, C405Y, A412R	PV135098
		–	1	U60986	130–423	T135Y, T150Y, T159Y, A162W, T168Y, T171Y, T210Y, T234Y, T240Y, T246Y, A261R, T267Y, T279Y, T303Y, T318Y, C327Y, A333M, C336T, T342Y, C360S, A393R, T396Y, C405Y, A412R	PV135099
Cat	A	AI	1	L40509	78–481	None	PV135100
	A	AII	1	L40510	124–482	A223G	PV135101
	B	BIII	2	AF069059	42–389	C309T	PV135102
	D	–	1	U60986	78–480	C375T	PV135103
	F	–	3	AF069057	79–482	None	PV135104
		–	1	AF069057	79–485	G289R	PV135105

M: C/A; R: A/G; S: G/C; W: A/T; Y: C/T.

**Table 3:** Frequency and molecular diversity of *Giardia duodenalis* identified at the *bg* locus in the canine and feline populations (GenBank accession numbers are provided).

Host	Assemblage	No. isolates	Reference sequence	Stretch	Single nucleotide polymorphisms	GenBank ID
Dog	B	1	AY072727	155–569	C309Y	PV135041
	C	2	AY545646	11–504	None	PV135042
		1	AY545646	11–504	G136R, C217Y	PV135043
		1	AY545646	55–500	C217T	PV135044
		1	AY545646	55–430	C217Y	PV135045
		1	AY545646	15–511	C217T, G364A	PV135046
		1	AY545646	101–510	T246Y	PV135047
		1	AY545646	6–511	A419R	PV135048
	D	3	AY545647	151–573	None	PV135049
		2	AY545647	103–596	A159G	PV135050
		1	AY545647	99–590	A159R, A201G	PV135051
		3	AY545647	103–594	A201G	PV135052
		2	AY545647	98–654	A201G, C207Y	PV135053
		1	AY545647	155–594	A201R, C207Y	PV135054
		1	AY545647	104–742	A201G, T368W	PV135055
		1	AY545647	103–594	A201G, C207Y, C561Y	PV135056
		1	AY545647	103–593	A201R, C561Y	PV135057
		1	AY545647	102–590	233DelA, A397W, G517R	PV135058
Cat	F	1	AY647264	156–453	None	PV135059
		1	AY647264	C102–594	C123T, G159T	PV135060

R: A/G; W: A/T; Y: C/T.

### 3.2 Molecular characterization of *Cryptosporidium* spp. isolates

Four *Cryptosporidium* species were identified in canine samples (Table 4).

**Table 4:** Frequency and molecular diversity of *Cryptosporidium* spp. identified in the canine and feline populations investigated in the present study. GenBank accession numbers are provided.

Host	Species	Genotype	No. isolates	Locus	Reference sequence	Stretch	Single nucleotide polymorphisms	GenBank ID
Dog	*C. canis*	–	7	*ssu* rRNA	AF112576	529–1021	None	PV123153
	*C. canis*	–	1	*ssu* rRNA	AF112576	534–1017	645DelT	PV123154
		XXe2	1	*gp60*	OQ787086	2–675	None	PV135061
	*C. felis*	–	1	*ssu* rRNA	AF108862	550–1029	None	PV123155
	*C. hominis*	–	1	*ssu* rRNA	AF108865	566–997	A703R, A781R, A885R	PV123156
	*C. parvum*	–	1	*ssu* rRNA	L16996	609–996	687DelA, A689T, A888G	PV123157
Cat	*C. felis*	–	7	*ssu* rRNA	AF108862	537–1057	None	PV123158
		–	1	*ssu* rRNA	AF108862	630–1055	698_700DelTTT	PV123159
	*C. parvum*	–	1	*ssu* rRNA	L16996	571–914	A851R	PV123160

Del: Deletion; *gp60*: 60 kDa glycoprotein; Ins: Insertion; R: A/G; *ssu* rRNA: Small subunit ribosomal RNA.

**Fig. 1:**
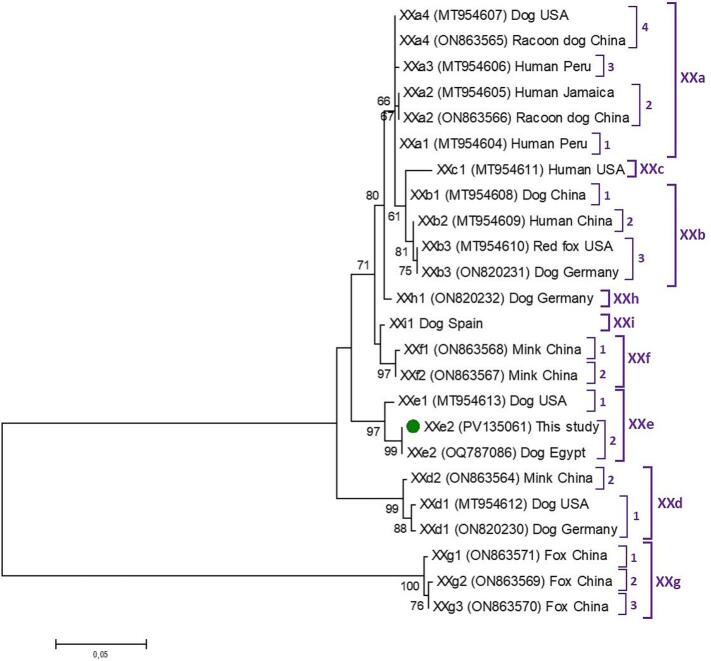
Phylogenetic relationship among nine *Cryptosporidium canis* subtype families (XXa–XXi) revealed by a maximum likelihood analysis of the partial *gp60* gene. Substitution rates were calculated by using the general time reversible model. Numbers on branches are percent bootstrapping values over 50% using 1000 replicates. The filled green circle indicates the nucleotide sequence of the subtype XXe2 generated in the present study. (For interpretation of the references to colour in this figure legend, the reader is referred to the web version of this article.)

*C. canis* was the predominant species (*n* = 8), whereas *C. felis*, *C. hominis* and *C. parvum* were detected sporadically (*n* = 1 each). A single *C. canis* isolate was successfully characterized at the *gp60* locus. Phylogenetic analysis of this *gp60* sequence confirmed its placement within the *C. canis* subtype family XXe (Fig. 1) and was designated as XXe2 according to the current subtype nomenclature (Xiao and Feng, 2017). The remaining *Cryptosporidium* isolates could not be subtyped at this marker.

In cats, two species were identified, including feline-adapted *C. felis* (*n* = 7) and zoonotic *C. parvum* (*n* = 1) (Table 4).

## 4 Discussion

Molecular epidemiological data on *G. duodenalis* and *Cryptosporidium* spp. in companion animals in Spain remain relatively limited, particularly in cats. Within this context, the present study provides additional molecular evidence on the frequency and diversity of species/genotypes circulating in dogs and cats in Madrid, central Spain.

In dogs, most *G. duodenalis* infections corresponded to host-adapted assemblages C and D, either as single or mixed infections. This result is consistent with those from previous epidemiological studies conducted in Spain (Dado et al., 2012) and other European countries (Ryan and Cacciò, 2013; Feng and Xiao, 2011). In cats, host-adapted assemblage F was the most frequently detected genotype, in agreement with previous studies identifying this assemblage in felids (Monis et al., 2009; Ryan and Cacciò, 2013). Nevertheless, the number of feline isolates successfully genotyped was relatively small. Therefore, these findings should be interpreted with caution.

Zoonotic *G. duodenalis* assemblages A and B were detected in both populations, with frequencies of 3.1% and 4.6% in dogs, respectively, and 20.0% for both in cats. Although these assemblages are commonly reported in human infections, the role of dogs and cats as an important source of human giardiosis remains unclear, and pets cannot be conclusively excluded as direct zoonotic transmission between pets and their owners (Ballweber et al., 2010; Ryan and Cacciò, 2013).

Remarkably, many of the single nucleotide polymorphisms observed in canine-adapted assemblage C and D sequences corresponded to ambiguous (double-peak) positions upon chromatogram inspection. These double peaks may reflect either true mixed infections involving distinct genetic variants of the parasite or allelic sequence heterozygosity (ASH), arising from the presence of divergent alleles within a single *G. duodenalis* isolate. Importantly, ASH has been demonstrated at the single-cell level in assemblage B lineages (Ankarklev et al., 2012), indicating that such genetic variability is intrinsic to individual parasites rather than solely attributable to co-infections. In dog-adapted assemblages C and D, relatively high levels of ASH (≈0.7–0.9%) have been reported (Kooyman et al., 2019), suggesting substantial intra-isolate genetic diversity that may be linked to host adaptation and evolutionary processes. ASH is particularly relevant in molecular epidemiological studies, as it contributes to the sequence heterogeneity frequently observed in multilocus genotyping, thereby complicating assemblage and sub-assemblage assignment and potentially obscuring transmission patterns (Cacciò and Sprong, 2010). Conversely, ASH also provides valuable insights into parasite population structure, recombination processes, and the genetic diversity underlying host specificity.

Regarding *Cryptosporidium*, several species were detected in dogs, with *C. canis* causing most infections. This canine-adapted *Cryptosporidium* species is the most prevalent *Cryptosporidium* species identified in dogs globally (Ryan et al., 2021). One *C. canis* isolate was successfully subtyped at the *gp60* locus and classified as subtype XXe2, a member of the *gp60* family XX initially described by Xiao and Feng (2017). This information contributes to expanding current knowledge on the epidemiology of *C. canis*, as limited data are available on the genetic diversity of this species at the *gp60* locus.

The lower genotyping success observed for *G. duodenalis* compared to *Cryptosporidum* spp. is consistent with the well-recognised methodological challenges associated with the molecular detection of enteric protozoa directly from faecal samples. Faecal material frequently contains PCR inhibitors, including complex polysaccharides, bile salts and other organic compounds that can reduce amplification efficiency and compromise sequencing performance (Hawash, 2014; Koehler et al., 2014). In addition, inefficient DNA recovery from cysts and oocysts particularly in samples with low parasite burdens, represents an important limitation for successful molecular characterization (Nantavisai et al., 2007; Paulos et al., 2016).

Amplification success in *G. duodenalis* has been reported to vary substantially depending on sample quality, storage conditions, DNA extraction procedures and molecular markers targeted (Elwin et al., 2014: Koehler et al., 2014). Furthermore, locus-dependent differences in PCR performance may contribute to incomplete multilocus genotyping results (Elwin et al., 2014). In contrast, *Cryptosporidum* spp. generally show higher amplification and sequencing success rates, likely due to the more consistent performance of commonly used targets such as the 18S rRNA gene and differences in template availability (Koehler et al., 2014; Hawash, 2014). Overall, the genotyping success rates obtained in the present study are consistent with those expected from routine diagnostic faecal samples and likely reflect inherent technical limitations rather than analytical bias.

The identification of primarily anthroponotic *C. hominis* and zoonotic *C. parvum* in two dogs raises the question of whether this host can serve as a source of human cryptosporidiosis. This possibility should be considered; however, it is also likely that these findings correspond to spurious infections due to mechanical carriage. Similar results have been reported sporadically in dogs in previous studies (Ng et al., 2011; Gil et al., 2017; Barbosa et al., 2023). In cats, most infections were caused by *C. felis*, while *C. parvum* was detected in a single sample, in agreement with the host preference described for these species (Ryan et al., 2021). Although these results confirm the presence of potentially zoonotic *Cryptosporidium* species in companion animals, their low frequency suggests that dogs and cats are unlikely to play a major role as reservoirs for human cryptosporidiosis in Spain (de Lucio et al., 2017).

Interestingly, several atypical host-genotype associations were observed, including the detection of feline-adapted *G. duodenalis* assemblage F in a dog and canine-adapted assemblage D in a cat. Similar cross-species detections have occasionally been reported in environments where different animal species coexist in proximity and may reflect sporadic cross transmission events or environmental contamination (Ryan and Cacciò, 2013). Again, mechanical carriage may explain some of these findings.

From a public and environmental health perspective, the detection of zoonotic *G. duodenalis* assemblages and *Cryptosporidium* species in companion animals indicates that pets may contribute, although probably to a limited extent, to the environmental dissemination of infective stages. As cysts and oocysts of these parasites are environmentally resistant and can persist for prolonged periods in water and soil, they can facilitate circulation across human, animal and environmental interfaces. Consequently, the presence of these parasites in companion animal remains relevant within a One Health framework and should not be underestimated, particularly in settings where close contact between humans and pets is common (Feng and Xiao, 2011; Ryan et al., 2021).

This study has some limitations that should be acknowledged. First, long-term storage of DNA samples before molecular testing might have reduced the performance of the PCR assays as consequence of suboptimal DNA quantity and/or quality. And second, the canine and feline DNA panels analysed were obtained from animals geographically restricted to Madrid; therefore, the results generated here might not be fully representative of the epidemiological situation present in canine and feline populations from other Spanish regions.

## 5 Conclusions

This study provides molecular data on *Giardia duodenalis* and *Cryptosporidium* spp. circulating in dogs and cats from Madrid. Most infections were caused by host-adapted genotypes, whereas zoonotic assemblages and species were detected only sporadically and confirm that companion animals do not seem to play a major role as source of human infections. From a One Health perspective, the presence of environmentally resistant cysts and oocysts highlights the potential contribution of companion animals to environmental contamination pathways relevant to waterborne and foodborne transmission. Further studies including environmental sampling would help to better clarify the role of companion animals in the transmission dynamics of these intestinal parasites.

## CRediT authorship contribution statement

**Juan P. Barrera:** Methodology, Investigation, Formal analysis, Writing – original draft. **Ana Montoya:** Supervision, Investigation, Formal analysis, Writing – review & editing. **Aida de Lucio:** Methodology. **Pamela C. Köster:** Formal analysis. **Begoña Bailo:** Formal analysis. **David Carmena:** Supervision, Methodology, Investigation, Data curation, Writing – review & editing. **Guadalupe Miró:** Supervision, Investigation, Conceptualization, Writing – review & editing.

## Declaration of competing interest

The authors have declared no conflict of interest.

## Data Availability

The data that support the findings of this study are available within the main body of the manuscript.
