# Supplementary material for: Molecular characterization and zoonotic potential of Giardia and Cryptosporidium infections in dogs and cats in Central Spain
Source: Food Waterborne Parasitol. 2026 Jun 17;44:e00351. doi: 10.1016/j.fawpar.2026.e00351 (PMC13315442; doi:10.1016/j.fawpar.2026.e00351)
Supplement: Supplementary file 1 — Supplementary material 1 [file mmc1.docx]

**Table S1**. Diversity and frequency of *Giardia duodenalis* assemblages in canine and feline populations in Spain, 2012–2023.

|  |  |  |  |  | **Assemblage** | | | | | |  |
| --- | --- | --- | --- | --- | --- | --- | --- | --- | --- | --- | --- |
| **Host species** | **Region** | **Isolates (*n*)** | **Method** | **Loci** | **A** | **B** | **C** | **D** | **F** | **Mixed (*n*)** | **References** |
| Dog | Álava | 3 | PCR+SS | *bg*, *gdh* | 0 | 0 | 3 | 0 | 0 | – | de Lucio et al. (2017) |
|  | Álava | 19 | PCR+SS | *bg*, *gdh* | 7 | 8 | 3 | 1 | 0 | – | Gil et al. (2017) |
|  | Catalonia | 6 | PCR+SS | *bg*, *gdh*, *tpi* | 0 | 0 | 1 | 4 | 0 | C+D (1) | Ortuño et al. (2014) |
|  | Castellón | 35 | PCR+SS | *bg*, *gdh* | 5 | 8 | 2 | 13 | 0 | A+B (4), A+D (1), A+B+D (2) | Adell-Aledón et al. (2018) |
|  | Madrid | 63 | PCR-RFLP | *bg*, *gdh* | 3 | 22 | 1 | 7 | 0 | A+B (3), A+C (3), A+E (1), B+C (3), B+D (11), B+E (4), C+D (1), C+E (1), B+C+D (3) | Dado et al. (2012) |
|  | Madrid | 10 | PCR+SS | *bg*, *gdh*, *tpi* | 1 | 0 | 4 | 4 | 0 | C+D (1) | Mateo et al. (2023) |
| **Total** |  |  |  |  | **16** | **38** | **14** | **29** | **0** | **39** |  |
| Cat | Álava | 2 | PCR+SS | *bg*, *gdh* | 0 | 0 | 0 | 0 | 2 | – | de Lucio et al. (2017) |
|  | Álava | 2 | PCR+SS | *bg*, *gdh* | 1 | 0 | 0 | 0 | 1 | – | Gil et al. (2017) |
|  | Madrid | 1 | PCR-RFLP | *bg*, *gdh* | 0 | 0 | 0 | 0 | 0 | A+F (1) | Dado et al. (2012) |
| **Total** |  |  |  |  | **1** | **0** | **0** | **0** | **3** | **1** |  |

*bg*: β-giardin; *gdh*: Glutamate dehydrogenase; PCR: Polymerase chain reaction; RFLP: Restriction fragment length polymorphism; SS: Sanger sequencing; *tpi*: triose phosphate isomerase.
