# Supplementary material for: Molecular characterization and zoonotic potential of Giardia and Cryptosporidium infections in dogs and cats in Central Spain
Source: Food Waterborne Parasitol. 2026 Jun 17;44:e00351. doi: 10.1016/j.fawpar.2026.e00351 (PMC13315442; doi:10.1016/j.fawpar.2026.e00351)
Supplement: Supplementary file 2 — Supplementary material 2 [file mmc2.docx]

**Table S2**. Diversity and frequency of *Cryptosporidium* species in canine and feline populations in Spain, 2017–2023).

|  |  |  |  |  | ***Cryptosporidium* species** | | | | |  |
| --- | --- | --- | --- | --- | --- | --- | --- | --- | --- | --- |
| **Host species** | **Region** | **Isolates (*n*)** | **Method** | **Loci** | ***C. canis*** | ***C. felis*** | ***C. hominis*** | ***C. parvum*** | **Mixed (*n*)** | **References** |
| Dog | Álava | 2 | PCR+SS | *ssu* RNA | 2 | 0 | 0 | 0 | – | de Lucio et al. (2017) |
|  | Álava | 6 | PCR+SS | *ssu* RNA | 5 | 0 | 1 | 0 | – | Gil et al. (2017) |
|  | Madrid | 5 | PCR+SS | *ssu* RNA | 4 | 0 | 0 | 1 | – | Mateo et al. (2023) |
| **Total** |  |  |  |  | **11** | **0** | **1** | **1** | **0** |  |
| Cat | Álava | 1 | PCR+SS | *ssu* RNA | 0 | 1 | 0 | 0 | – | Gil et al. (2017) |
|  | Álava | 1 | PCR+SS | *ssu* RNA | 0 | 1 | 0 | 0 | – | de Lucio et al. (2017) |
| **Total** |  |  |  |  | **0** | **2** | **0** | **0** | **0** |  |

*ssu* rRNA: Small subunit ribosomal RNA.
