# Supplementary material for: Molecular characterization and zoonotic potential of Giardia and Cryptosporidium infections in dogs and cats in Central Spain
Source: Food Waterborne Parasitol. 2026 Jun 17;44:e00351. doi: 10.1016/j.fawpar.2026.e00351 (PMC13315442; doi:10.1016/j.fawpar.2026.e00351)
Supplement: Supplementary file 3 — Supplementary material 3 [file mmc3.docx]

**Table S3.** PCR cycling conditions used for the molecular confirmation and characterization of the protozoan parasites of canine and feline origin.

|  |  | **Temperature and time** | | | |  |  |  |
| --- | --- | --- | --- | --- | --- | --- | --- | --- |
| **Target organism** | **Locus** | **Initial denaturation** | **Denaturation** | **Annealing** | **Extension** | **No. cycles** | **Final extension** | **References** |
| *Giardia duodenalis* | *ssu* rRNA | 95°C 15 min | 95°C 15 s | 60°C 1 min | 72°C 30 s | 45 | – | Verweij et al. (2003) |
|  | *gdh* | 95°C 3 min | 95°C 30 s | 55°C 30 s | 72°C 1 min | 35 | 72°C 7 min | Read et al. (2004) |
|  | *bg* | 95°C 7 min | 95°C 30 s | 65/55°C 30 s | 72°C 1 min | 35 | 72°C 7 min | Lalle et al. (2005) |
| *Cryptosporidium* spp. | *ssu* rRNA | 94°C 3 min | 94°C 40 s | 50°C 40 s | 72°C 1 min | 35 | 72°C 10 min | Tiangtip and Jongwutiwes (2002) |
| *Cryptosporidium canis* | *gp60* | 94°C 5 min | 94°C 45 s | 52°C 45 s | 72°C 80 s | 35 | 72°C 10 min | Jiang et al. (2021) |
| *Cryptosporidium hominis/parvum* | *gp60* | 94°C 5 min | 94°C 45 s | 59/50°C 45 s | 72°C 1 min | 35 | 72°C 10 min | Feltus et al. (2006) |

*bg*: β-giardin; *gdh*: Glutamate dehydrogenase; *gp60*: 60 kDa glycoprotein; *ssu* rRNA: Small subunit ribosomal RNA.
