# Supplementary material for: Molecular characterization and zoonotic potential of Giardia and Cryptosporidium infections in dogs and cats in Central Spain
Source: Food Waterborne Parasitol. 2026 Jun 17;44:e00351. doi: 10.1016/j.fawpar.2026.e00351 (PMC13315442; doi:10.1016/j.fawpar.2026.e00351)
Supplement: Supplementary file 4 — Supplementary material 4 [file mmc4.docx]

**Table S4.** Oligonucleotides used for the molecular confirmation and characterization of the protozoan parasites of canine and feline origin.

| **Target organism** | **Locus** | **Oligonucleotide** | **Sequence (5´–3´)** | **Generated amplicon (bp)** | **References** |
| --- | --- | --- | --- | --- | --- |
| *Giardia duodenalis* | *ssu* rRNA | Probe | FAM–CCCGCGGCGGTCCCTGCTAG–BHQ1 | 62 | Verweij et al. (2003) |
|  |  | Gd-80F | GACGGCTCAGGACAACGGTT |  |  |
|  |  | Gd-127R | TTGCCAGCGGTGTCCG |  |  |
|  | *gdh* | GDHeF | TCAACGTYAAYCGYGGYTTCCGT | 432 | Read et al. (2004) |
|  |  | GDHiF | CAGTACACCTCYGCTCTCGG |  |  |
|  |  | GDHiR | GTTRTCCTTGCACATCTCC |  |  |
|  | *bg* | G7_F | AAGCCCGACGACCTCACCCGCAGTGC | 511 | Cacciò et al. (2002) |
|  |  | G759_R | GAGGCCGCCCTGGATCTTCGAGACGAC |  |  |
|  |  | G99_F | GAACGAACGAGATCGAGGTCCG |  | Lalle et al. (2005) |
|  |  | G609_R | CTCGACGAGCTTCGTGTT |  |  |
| *Cryptosporidium* spp. | *ssu* rRNA | CR-P1 | CAGGGAGGTAGTGACAAGAA | 587 | Tiangtip and Jongwutiwes (2002) |
|  |  | CR-P2 | TCAGCCTTGCGACCATACTC |  |  |
|  |  | CR-P3 | ATTGGAGGGCAAGTCTGGTG |  |  |
|  |  | CPB-DIAGR | TAAGGTGCTGAAGGAGTAAGG |  |  |
| *Cryptosporidium canis* | *gp60* | GP60 Ccanis_F1 | ATACTCTGGTCTCCCGTTT | 700 | Jiang et al. (2020) |
|  |  | GP60 Ccanis_R1 | GTACTCGGAAGCGGTGTA |  |  |
|  |  | GP60 Ccanis_F2 | AAGGCGCCTCACTCATT |  |  |
|  |  | GP60 Ccanis_R2 | TCAGTTAGATATCACCCATTAA |  |  |
| *Cryptosporidium hominis*/*parvum* | *gp60* | AL-3531 | ATAGTCTCCGCTGTATTC | 830 | Feltus et al (2006) |
|  |  | AL-3535 | GGAAGGAACGATGTATCT |  |  |
|  |  | AL-3532 | TCCGCTGTATTCTCAGCC |  |  |
|  |  | AL-3534 | GCAGAGGAACCAGCATC |  |  |

*bg*: β-giardin; *gdh*: Glutamate dehydrogenase; *gp60*: 60Kda glycoprotein; *ssu* rRNA: Small subunit ribosomal RNA.
