# Supplementary material for: Molecular characterization and zoonotic potential of Giardia and Cryptosporidium infections in dogs and cats in Central Spain
Source: Food Waterborne Parasitol. 2026 Jun 17;44:e00351. doi: 10.1016/j.fawpar.2026.e00351 (PMC13315442; doi:10.1016/j.fawpar.2026.e00351)
Supplement: Supplementary file 5 — Supplementary material 5 [file mmc5.docx]

**Table S5.** Distribution of *G. duodenalis* genotypes and *Cryptosporidium* species according to host species and analysed variables.

|  |  |  |  | **Origin** | | | **Age (years)** | | **Sex** | |
| --- | --- | --- | --- | --- | --- | --- | --- | --- | --- | --- |
| **Host** | **Pathogen** | **Genotype** | **No.** | **Owned** | **Breeding** | **Shelter** | **< 1** | **≥ 1** | **Male** | **Female** |
| Dog | *G. duodenalis* | AI | 2 | 0 | 0 | 2 | 0 | 2 | 1 | 1 |
|  |  | B/C | 1 | 0 | 1 | 0 | 0 | 1 | 0 | 1 |
|  |  | BIII | 1 | 0 | 0 | 1 | 0 | 1 | 1 | 0 |
|  |  | BIV | 2 | 0 | 0 | 2 | 1 | 1 | 0 | 2 |
|  |  | BIV/D | 1 | 0 | 0 | 1 | 0 | 1 | 1 | 0 |
|  |  | C | 22 | 3 | 1 | 18 | 6 | 16 | 12 | 10 |
|  |  | C/D | 3 | 2 | 0 | 1 | 0 | 3 | 2 | 1 |
|  |  | D | 31 | 4 | 3 | 24 | 10 | 21 | 18 | 13 |
|  |  | D | 1 | 0 | 0 | 1 | 0 | 1 | 0 | 1 |
|  |  | F | 1 | 0 | 0 | 1 | 1 | 0 | 1 | 0 |
|  | *C. canis* | – | 8 | 4 | 1 | 3 | 1 | 7 | 7 | 1 |
|  | *C. felis* | – | 1 | 0 | 1 | 0 | 0 | 1 | 0 | 1 |
|  | *C. hominis* | – | 1 | 0 | 0 | 0 | 0 | 0 | 0 | 0 |
|  | *C. parvum* | – | 1 | 0 | 1 | 0 | 0 | 1 | 0 | 1 |
| Cat | *G. duodenalis* | AI | 1 | 0 | 1 | 0 | 1 | 0 | 0 | 1 |
|  |  | AII | 1 | 0 | 1 | 0 | 0 | 1 | 1 | 0 |
|  |  | BIII | 2 | 0 | 2 | 0 | 0 | 2 | 2 | 0 |
|  |  | D | 1 | 0 | 1 | 0 | 1 | 0 | 0 | 1 |
|  |  | F | 5 | 0 | 3 | 2 | 1 | 4 | 1 | 4 |
|  | *C. felis* | – | 8 | 1 | 3 | 4 | 2 | 6 | 3 | 5 |
|  | *C. parvum* | – | 1 | 1 | 0 | 0 | 0 | 1 | 1 | 0 |
